# Bioabsorbable Pins for Treatment of Osteochondral Fractures of the Knee after Acute Patella Dislocation in Children and Young Adolescents

**DOI:** 10.1155/2012/249687

**Published:** 2012-06-14

**Authors:** A. Gkiokas, L. G. Morassi, S. Kohl, C. Zampakides, P. Megremis, D. S. Evangelopoulos

**Affiliations:** ^1^1st Department of Orthopaedic Surgery, P. & A. Kyriakou Children's Hospital, Athens, Greece; ^2^3rd Department of Orthopaedic Surgery, KAT Trauma Hospital, University of Athens, Greece; ^3^Department of Orthopaedic Surgery, University of Bern, Inselspital, Switzerland

## Abstract

A retrospective study was performed on the use of bioabsorbable pins in the fixation of osteochondral fractures (OCFs) after traumatic patellar dislocation in children. Eighteen children (13 females, 5 males) aged 11 to 15 years (mean age 13.1 years) with osteochondral fracture (OCF) of the knee joint were treated at the authors' institution. Followup ranged from 22 months to 5 years. Diagnosis was verified by X-ray and magnetic resonance imaging (MRI) of the knee and patella. In seven patients the osteochondral fragment was detached from the patella and in 11 it was detached from the lateral femoral condyle. All patients were subjected to open reduction and fixation of the lesion with bioabsorbable pins. Postoperatively, the knee was immobilized in a cast and all patients were mobilized applying a standardized protocol. Bone consolidation was successful in 17 of the 18 patients. Bioabsorbable pins reliably fix OCF in children and adolescents, demonstrating a high incidence of consolidation of the detached osteochondral fragment in short- and middle-term followup without requiring further operative procedures.

## 1. Introduction

Acute patellar dislocation is a common injury in early adolescence, with an incidence ranging from 29 to 42 per 100,000 children under 16 years of age [1, 2]. The etiology is mainly traumatic, involving either direct trauma to the knee or a twisting action. Adolescents, particularly those with preexisting ligamentous laxity of the knee, are prone to patellar dislocation. Osteochondral fractures (OCFs) of the patella or femur represent a major complication following patella dislocation, its incidence varying from 5% to 39% after dislocation [1]. The fractured fragments consist of both cartilaginous and bony parts. Certain anatomic variables may predispose to patellar lateral instability in adolescents, including patella alta, genu valgum, internal torsion of the femur, trochlear dysplasia, laxity of the medial patellofemoral ligament (MPFL), or increased Q angle [3]. The majority of lateral patellar dislocations occur at the initial stages of flexion of the knee joint, when the patella is not fully engaged in the femoral sulcus. In this phase, the MPFL acts as the primary restraint to the patella's lateral translation [2, 4].

Multiple surgical procedures have been described for the treatment of OCF. In the past, such lesions were regarded as loose bodies and were simply excised, leaving an area of bone devoid of cartilage. The absence of cartilage, especially on weight-bearing surfaces of the lateral condyle and the medial articular surface of the patella, predisposes patients to early osteoarthritis of the injured joint [5]. Several surgical techniques have been proposed for the treatment of the osteochondral defect, such as drilling, fixation of OCF by means of screws or pins, and allogeneic or autogenous osteochondral transplantation [6–8]. More recently the use of biodegradable pins has been introduced for the fixation of OCF [9]. The present study assesses the results of open reduction and internal fixation of OCF using biodegradable pins.

## 2. Patients and Methods

**Figure 1:**
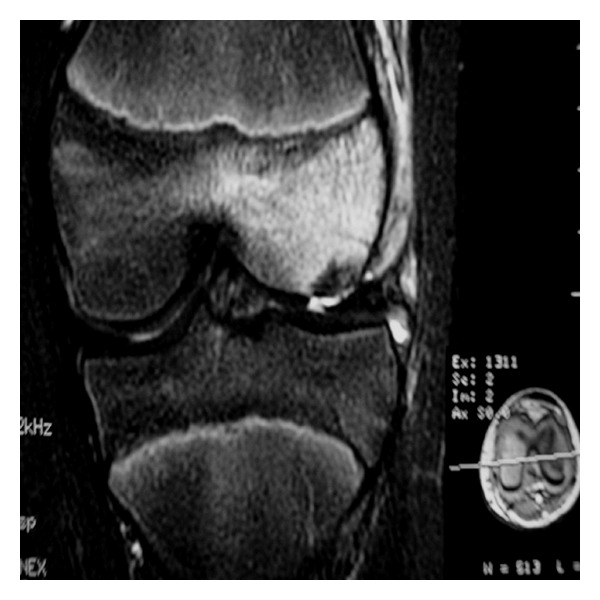
Preoperative MRI image of an osteochondral fracture of the lateral condyle in an 11-year-old girl.

Over a period of five years, 18 adolescents (5 males, 13 females; mean age 13.1 years, range 11–15 years) were treated at our institution (a level A trauma center) for osteochondral fracture following traumatic acute patellar dislocation. The mechanism of injury included either a direct blow to the patella or a twisting action. In two patients initial clinical evaluation found the patella to be dislocated with the knee flexed and swollen requiring immediate reduction. In the remaining 16 patients the patellar dislocation was reported by the patient, or caregivers, and spontaneous reduction with knee extension had occurred at the scene of injury. In all patients there was significant knee effusion, raising the suspicion of osteochondral damage secondary to traumatic patellar dislocation. Seven patients had intense hemarthroses that were treated by aspiration under local anesthesia. Thorough radiographic assessment of all 18 patients included anteroposterior, lateral, and tangential views of the knee. For the tangential view of the patella a Merchant's view was used. In one patient, a CT scan had been performed prior to transfer to our institution from a rural hospital. In the remaining 17 patients, preoperative MRI scans of the knee were performed to confirm the diagnosis, reveal the extent of cartilage damage, and rule out rupture of cruciate or collateral ligaments. All scans revealed bone marrow edema of the lateral femoral condyle (Figure 1).

**Figure 2:**
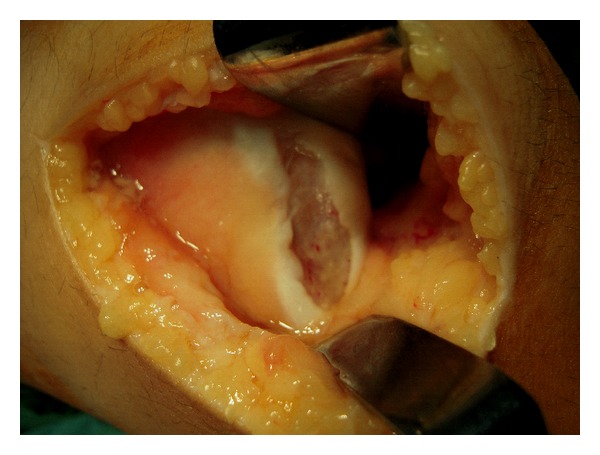
Image of the osteochondral lesion at the weight-bearing surface of the lateral condyle in the same patient.

**Figure 3:**
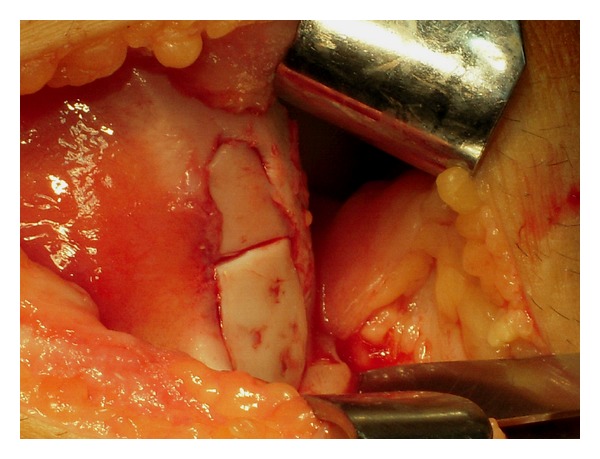
The same lesion after reduction and fixation of the two osteochondral fragments with five bioabsorbable pins.

All patients were treated surgically with open reduction and internal fixation of the osteochondral fragment with bioabsorbable pins (Arthrex Inc. Naples, Fl). The mean time from injury to surgical treatment was three days (range one to five days). Standard approach included a lateral parapatellar arthrotomy resulting in simultaneous release of the lateral retinaculum. The fracture location was identified and the osteochondral fragment was reduced to its origin (Figure 2). Fixation was achieved by inserting two to five (poly L-lactic acid) 1.3 mm pins (18 mm long) into a predrilled channel through the reduced fragments (Figure 3). A standardized rehabilitation protocol was applied for all 18 patients involving initial immobilization of the knee with a plaster cast for six weeks without weight-bearing. Six weeks postoperatively, the cast was removed and patients were allowed partial weight-bearing. Full weight-bearing was allowed after eight to nine weeks. Patients' rehabilitation was monitored monthly at the outpatient clinic evaluating quadriceps strength, range of motion (ROM) of the operated knee, and visual analogue scale (VAS) for pain, while the consolidation process was assessed using plain knee radiographs monthly after surgery for six months and knee MRI scan at 6, 12, 24, and 36 months after surgery.

## 3. Results 

All 18 patients were subjected to open reduction and internal fixation of the osteochondral fragment. The detached fragments were primarily chondral with small bony parts and surprisingly larger than expected in the majority of the patients. Seven (38%) of the detached fragments were from the medial facet of the patella, 11 (61%) from the lateral condyle of the femur on a weight-bearing surface.

**Figure 4:**
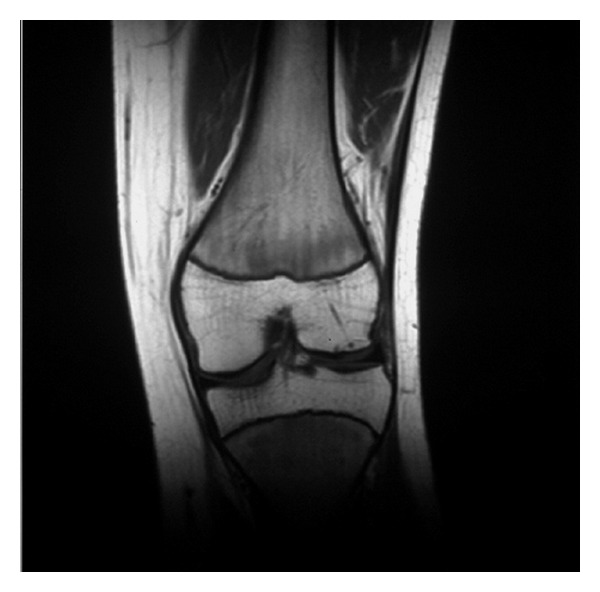
Postoperative MRI image showing consolidation of the osteochondral fragment and biodegradation of the pins in an 11-year-old patient at one-year followup.

The follow-up time after surgery varied from 22 months to 5 years. In one case the osteochondral fragment was detached from its origin causing pain and was removed arthroscopically eight weeks after surgery. In 17 patients in whom initial consolidation of the osteochondral fracture was achieved, the cast was removed at six-week followup and the mean range of flexion on the operated knee was 62° (range 10°–70°). A slight atrophy of the quadriceps muscle at the operated limb was observed in all 18 patients. Full strength of quadriceps muscle was restored two to four weeks after removal of the cast. Full ROM was equal in the two knees eight to 10 weeks after surgery. No sign of knee infection or foreign-body reaction was observed in any patient throughout the follow-up period. VAS for pain demonstrated significant improvement (from 8.6 before osteosynthesis to 1.2 after surgery, *P* < 0.005). Preoperative MRI scans revealed bone marrow edema of the lateral femoral condyle in most patients. Postoperative MRI scans showed consolidation of the osteochondral fragments with congruent joint surfaces and gradual degradation of the pins with concomitant gradual filling of the drill channels (Figure 4). Nevertheless, pin insertion sites were detectable on MRI scans at the three-year followup. In all 17 patients in whom consolidation was obtained, postoperative MRI scans demonstrated intact cartilage joint surface with small areas of cartilage thinning, but no areas of full-thickness loss.

## 4. Discussion

Acute patellar dislocation may result in OCF. In the past, OCFs following patellar dislocation were thought to be a rarity. In 1976 Rorabeck and Bobechko reported an incidence of 5% after acute patellar dislocation [10]. However, several recent studies report a higher incidence of OCF after patellar dislocation, ranging from 39% to 71% [1, 11–13]. As the aforementioned studies show, the incidence of such injuries had previously been underestimated. Nevertheless, despite the higher incidence of chondral injuries, not all injuries were displaced, requiring surgical treatment.

In the majority of cases of OCF with only one small bony fragment diagnosis is frequently missed. Plain radiographs often underestimate the size of the displaced fragments and therefore clinical signs such as hemarthrosis and the knowledge that such injuries are quite common is the key to proper diagnosis of OCF. Knee MRI scan or knee arthroscopy will confirm the diagnosis and may reveal the true extent of the cartilage damage.

The presence of OCF is a primary indication for surgical intervention after acute patellar dislocation. In the past, excision of the OCF was a predisposing factor for early degenerative arthritis [5]. Since then several materials have been developed for reliable fixation of the OCF fragments including metal pins and headless screws. The main disadvantage of these metal fixation devices is that they require removal after fragment consolidation. Microfracturing at the area of the lesion, osteochondral grafting, allogeneic, or autogenous osteochondral implantation, and autologous chondrocyte implantation have all been applied to repair such lesions [6–8]. Although good short-term results are reported, more studies with longer followup are needed to properly evaluate these techniques [6]. 

The introduction of biodegradable fixation devices was a revolution in the field of OCF fixation. Their main advantage is that they do not require removal. However, they are associated with a few complications, such as synovitis or foreign body reaction, as reported in the literature [14–16]. Poly L-lactic acid pins were applied for fixation of OCF fragments in the present study since they do not induce signs of inflammation or synovitis or other adverse reactions [9, 17, 18]. 

The postoperative follow-up period varied from 22 months to 5 years (mean time 34 months). The initial apparent consolidation of 17 of the 18 treated OCF lesions, seen postoperatively on plain radiographs, was confirmed by postoperative MRI findings (Figure 4). The reintegration rate using poly-L-lactic acid pins was very encouraging (94%) in the short- and middle-term followup. Follow-up MRI findings confirmed joint surface congruity and the vitality of the reintegrated OCF for up to 36 months after surgery. In all patients in whom reintegration was obtained, full ROM was restored and the knee joint was pain free. Another patellar dislocation was not reported in any patient. Radiological analysis demonstrated no major degenerative changes in the articular surface of the lateral condyle or the patella during postoperative followup.

## 5. Limitations

Despite our efforts to ensure validity, the present study displays certain limitations. This is a retrospective analysis and the number of patients composing the study group is limited to draw significant conclusions. Although our results demonstrate good functional outcome for all patients for a period ranging from 22 months to five years, a longer follow-up period is required to assess long-term changes of the articular surface of the lateral condyle.

## 6. Conclusion

The use of biodegradable pins for treatment of OCF in 18 children and early adolescents resulted in a high rate of consolidation, restoring congruity of the articular surfaces of the knee joint with no signs of foreign body reaction or synovitis. Studies with longer followup are still required to properly assess the benefits of bioabsorbable pins in the treatment of OCF.
